# Author Correction: Exosome-delivered EGFR regulates liver microenvironment to promote gastric cancer liver metastasis

**DOI:** 10.1038/s41467-023-37320-3

**Published:** 2023-03-27

**Authors:** Haiyang Zhang, Ting Deng, Rui Liu, Ming Bai, Likun Zhou, Xia Wang, Shuang Li, Xinyi Wang, Haiou Yang, Jialu Li, Tao Ning, Dingzhi Huang, Hongli Li, Le Zhang, Guoguang Ying, Yi Ba

**Affiliations:** 1 Tianjin Medical University Cancer Institute and Hospital, Tianjin Key Laboratory of Cancer Prevention and Therapy, National Clinical Research Center for Cancer, Tianjin, 300060 China; 2 Department of Gastroenterology, Tianjin First Center Hospital, Tianjin, 300192 China

Correction to: *Nature Communications* 10.1038/ncomms15016, published online 10 April 2017

The original Article contained an error made during the assembly of Figure 6E and 6G. In Fig 6E, the image shown in OE.NC group was misused from sh.NC group; while the OE.HGF panel was misused from a set of experiments performed at the same time for a different study. In Fig 6G, the image shown in OE.NC group was misused from sh.NC group; and the image shown in SGC exo+sh.HGF group was misused from the mock group. The corrected version of Figure 6 is shown below.


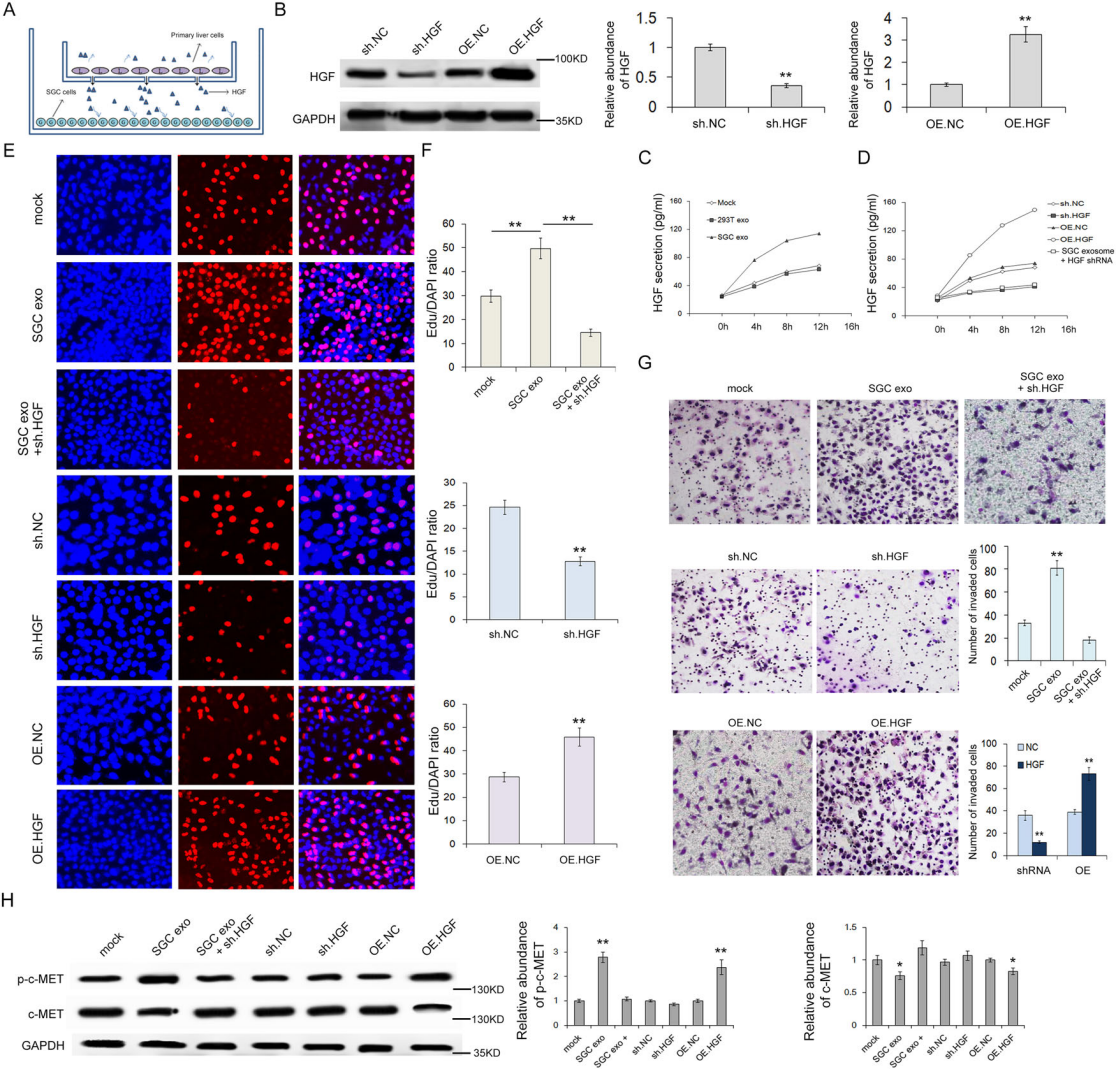


The figure has been corrected in the PDF and HTML versions of the Article.

Raw data are appended below.

## Supplementary information

Raw data

