# Supplementary material for: Exosome-delivered EGFR regulates liver microenvironment to promote gastric cancer liver metastasis
Source: Nat Commun. 2017 Apr 10;8:15016. doi: 10.1038/ncomms15016 (PMC5394240; doi:10.1038/ncomms15016)
Supplement: Supplementary Information — Supplementary Figures [file ncomms15016-s1.pdf]

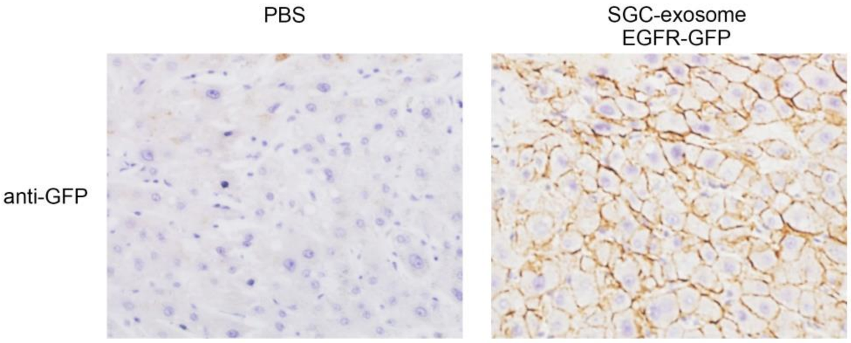

Supplementary Figure 1. Exosome-EGFR can be located in the cyto-membrane of liver in vivo. 60 μg SGC exosomes containing EGFR-GFP were injected into mice every two days via tail vein injection, and liver tissues were collected and analyzed at the 6th day.

Fig.1B

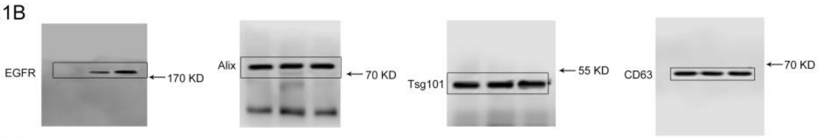

Fig.1C

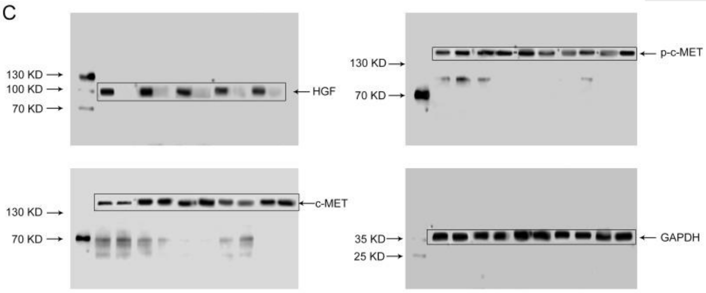

Fig.2B

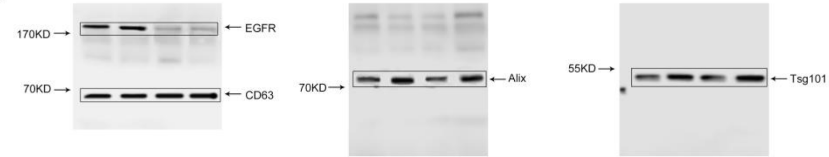

Fig.3A

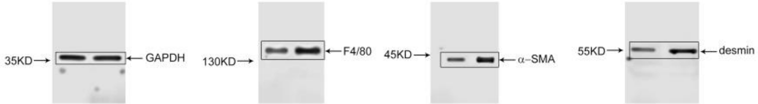

Fig.4A

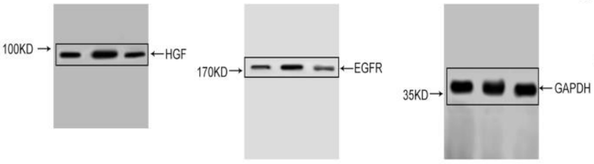

Fig.4C

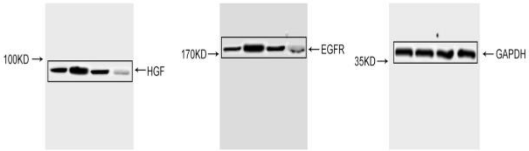

Fig.5C

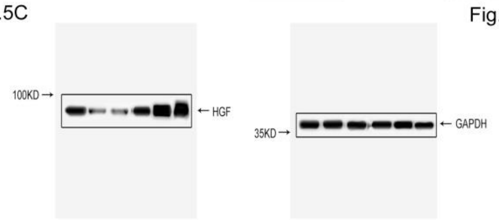

Fig.6C

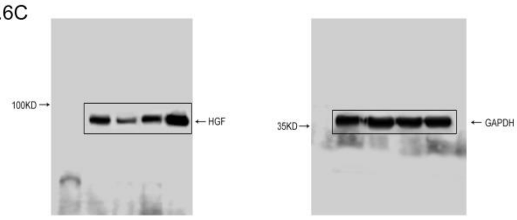

Fig.6H

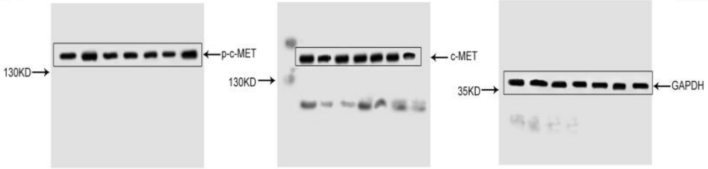

Fig.7C

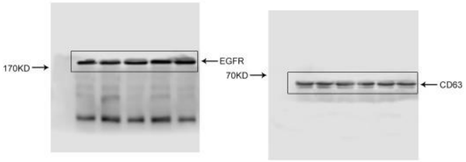

Fig.7D

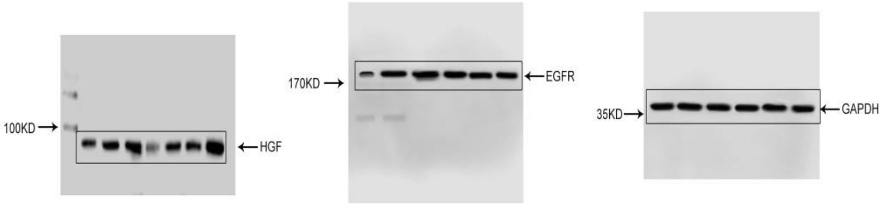

Fig.8H

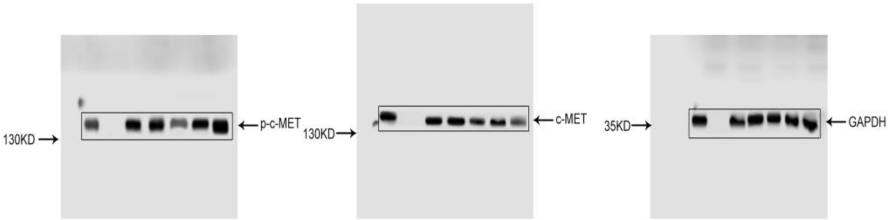

Supplementary Figure 2. Uncropped data. The uncropped illustrations used to prepare the main figures of this manuscript are shown with reference to their specific sub-figures indicated on each panel.
