# Supplementary material for: Author Correction: Exosome-delivered EGFR regulates liver microenvironment to promote gastric cancer liver metastasis
Source: Nat Commun. 2023 Mar 27;14:1700. doi: 10.1038/s41467-023-37320-3 (PMC10042995; doi:10.1038/s41467-023-37320-3)

Raw data of Fig 6G

mock

Data shown in Fig 6G

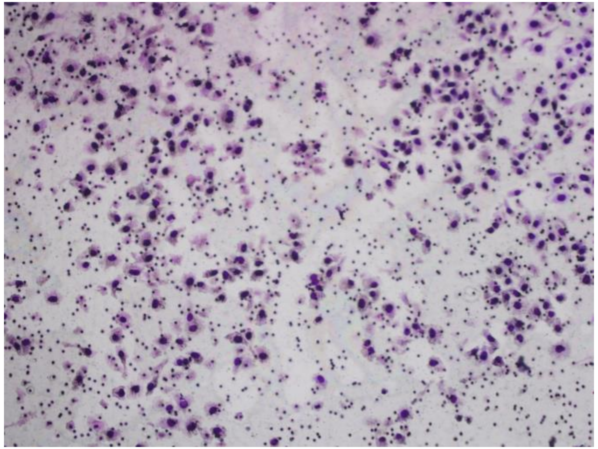

Data not shown

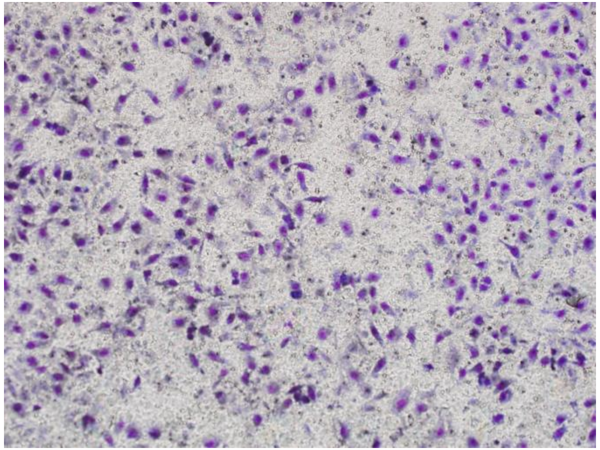

Data not shown

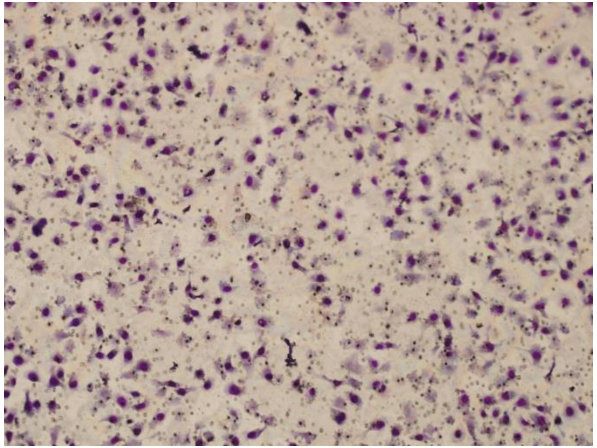

Raw data of Fig 6G

SGC exo

Data shown in Fig 6G

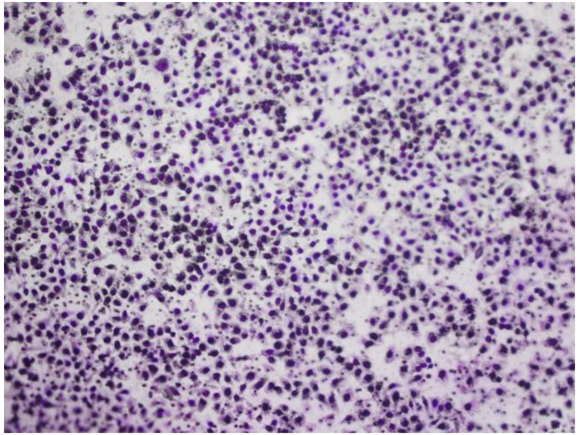

Data not shown

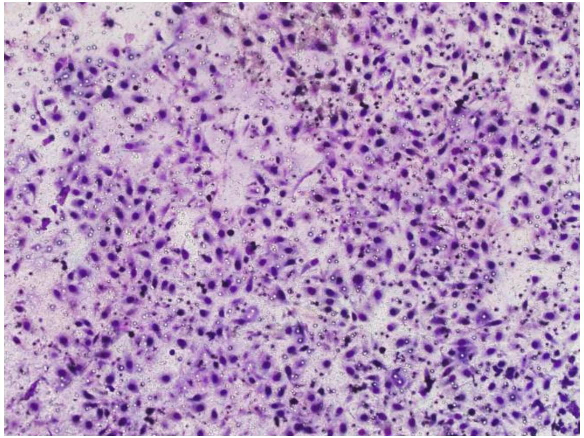

Data not shown

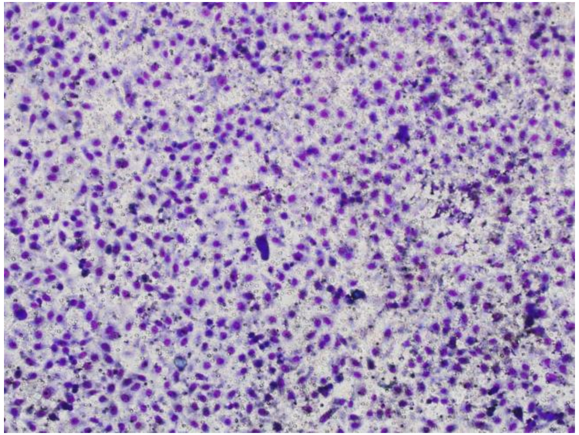

Raw data of Fig 6G

SGC exo + sh.HGF

Data shown in Fig 6G

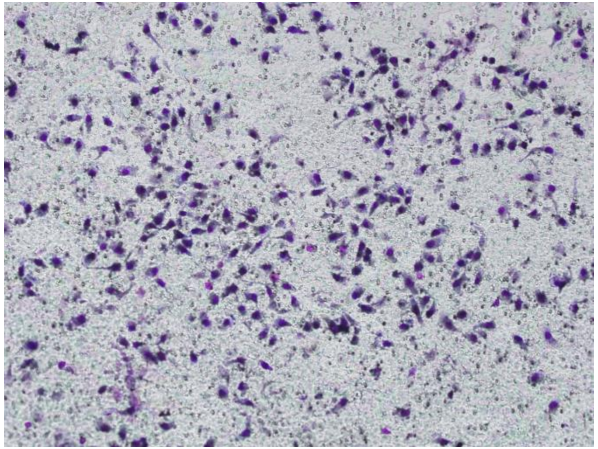

Data not shown

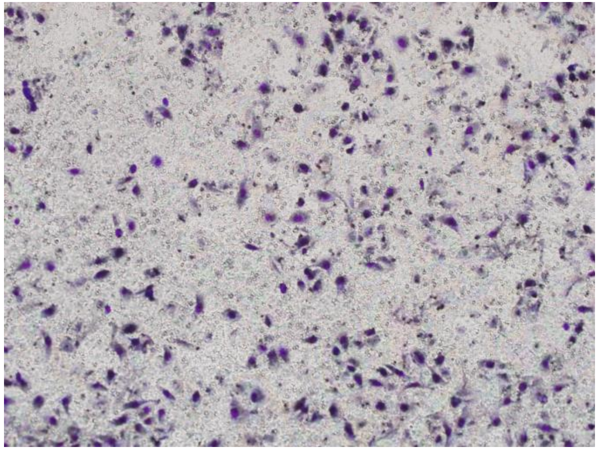

Data not shown

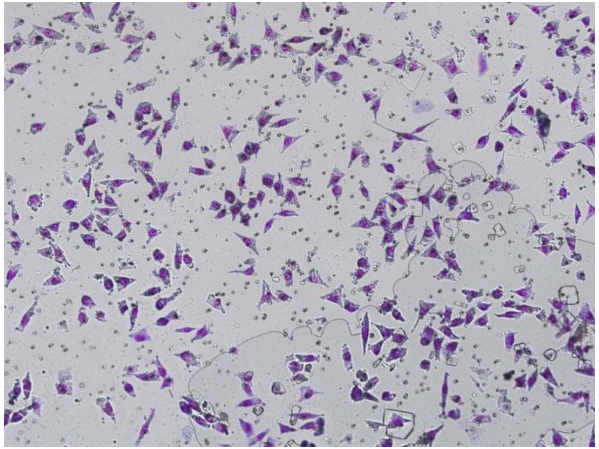

Raw data of Fig 6G

sh.NC

Data shown in Fig 6G

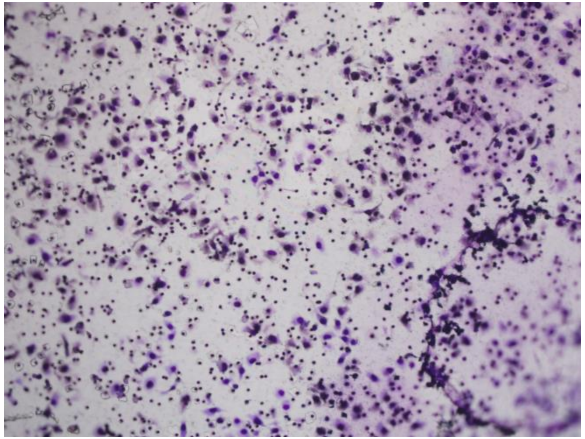

Data not shown

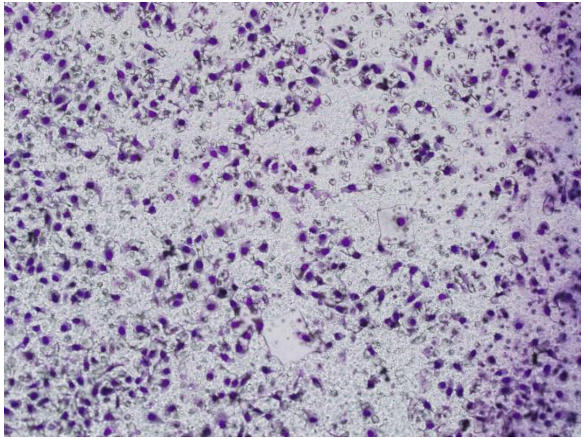

Data not shown

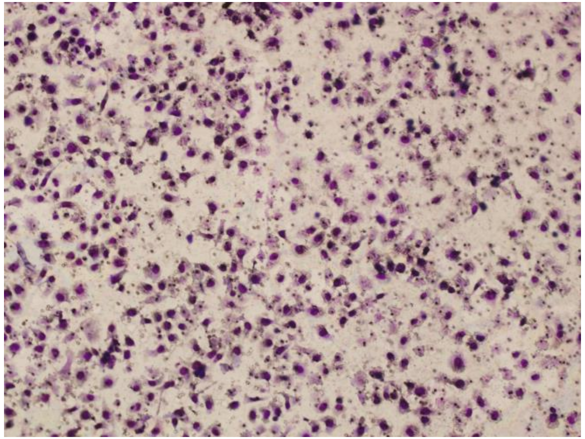

Raw data of Fig 6G

sh.HGF

Data shown in Fig 6G

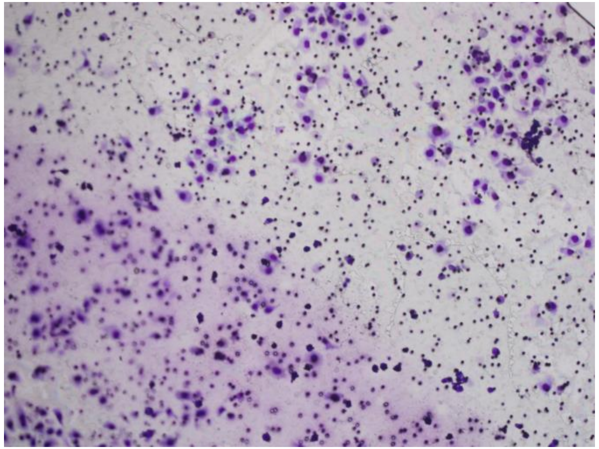

Data not shown

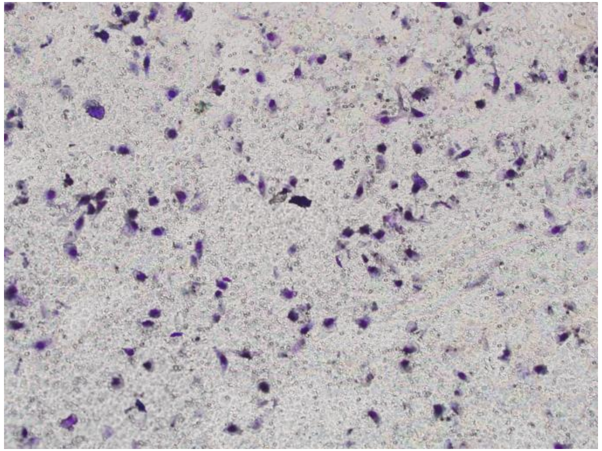

Data not shown

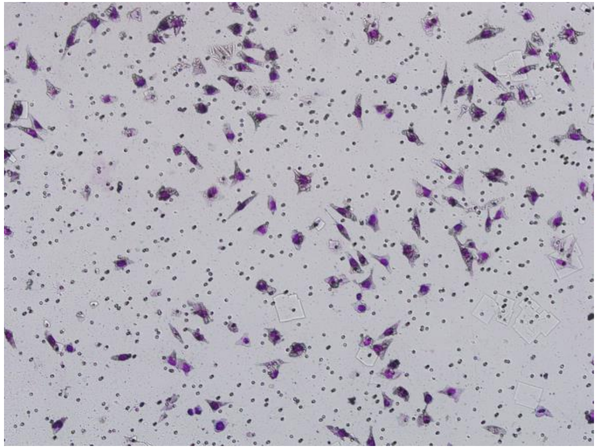

Raw data of Fig 6G

OE.NC

Data shown in Fig 6G

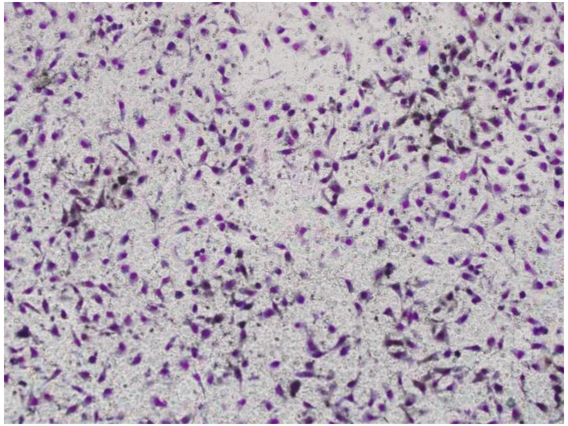

Data not shown

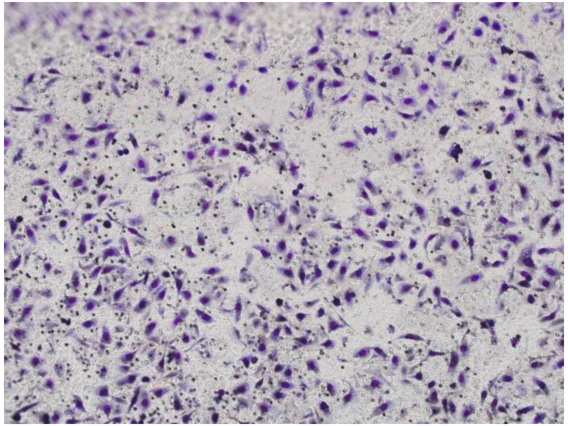

Data not shown

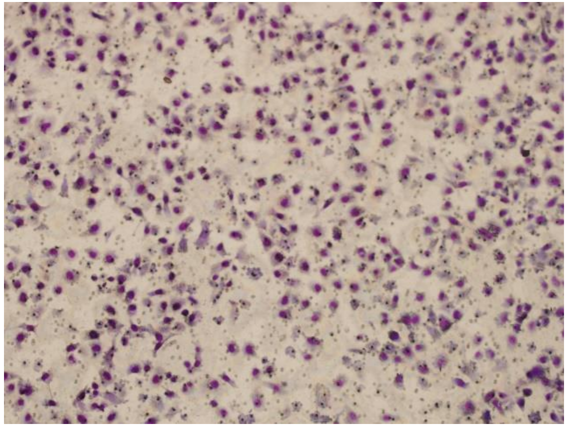

Raw data of Fig 6G

OE.HGF

Data shown in Fig 6G

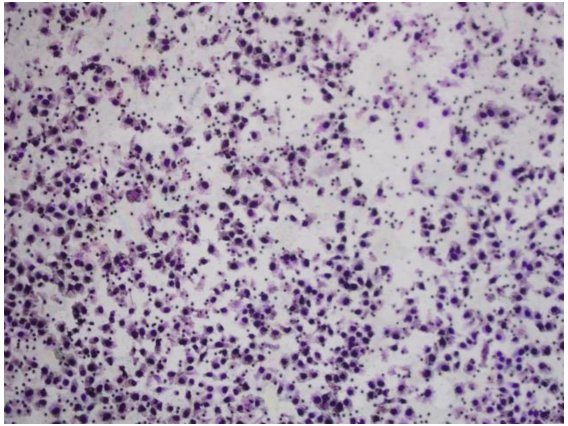

Data not shown

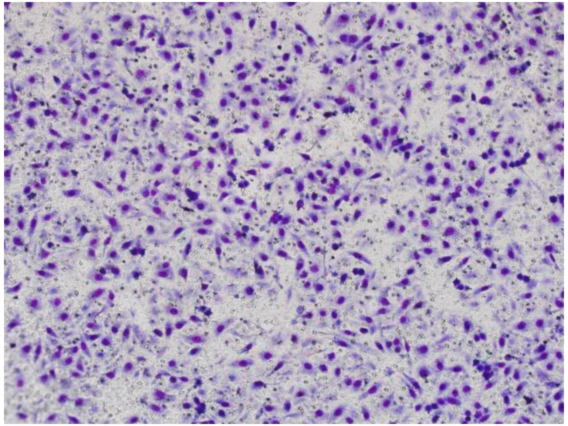

Data not shown

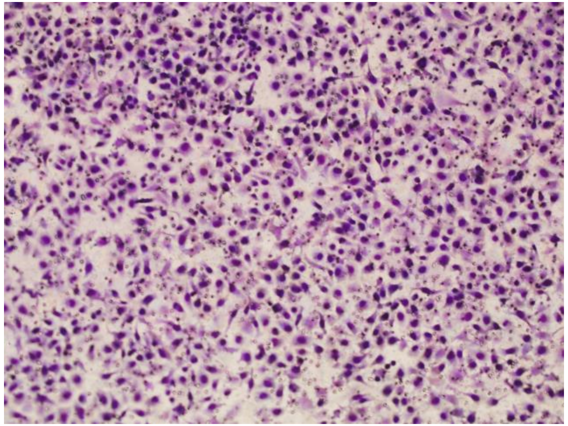

Raw data of Fig 6E

sh.NC

Data shown in Fig 6G | Data not shown | Data not shown

DAPI

EDU

Merge

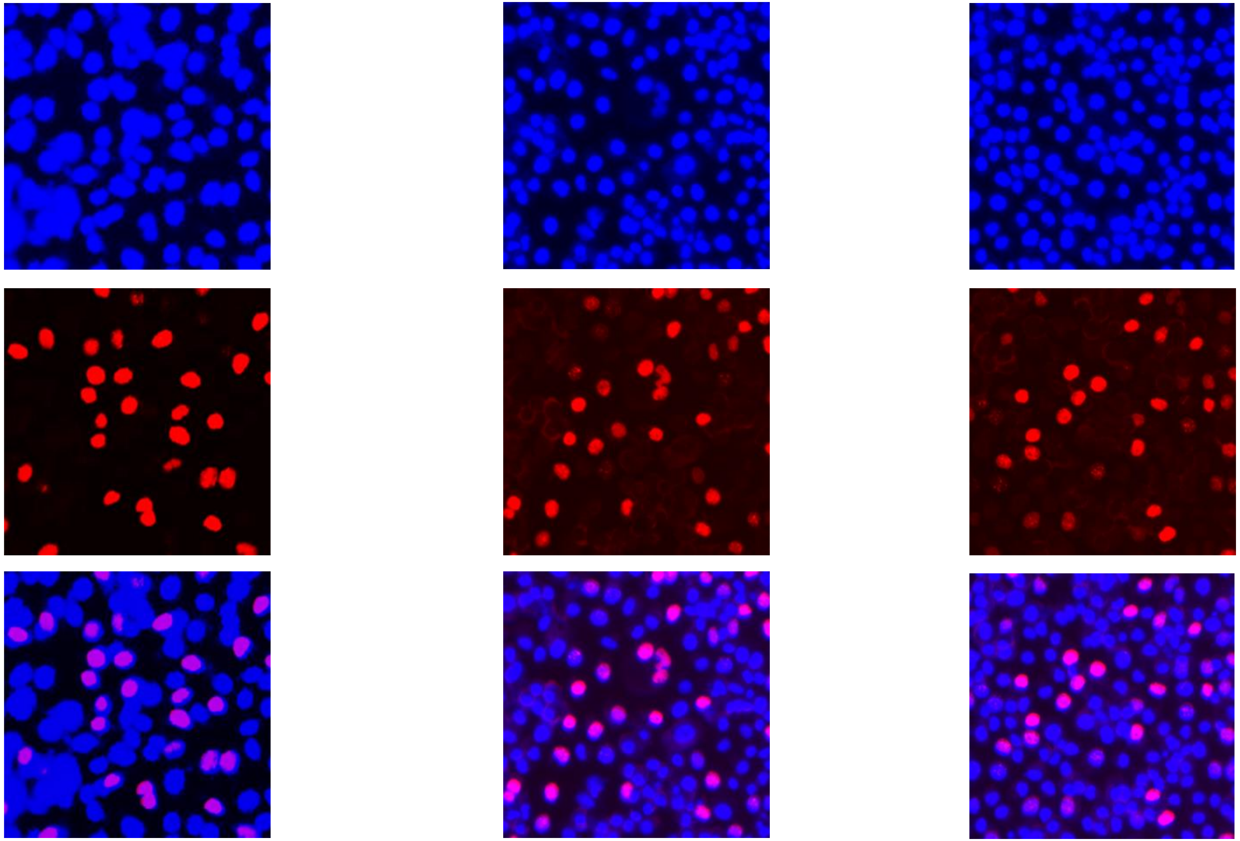

Raw data of Fig 6E

sh.HGF

Data shown in Fig 6G | Data not shown | Data not shown

DAPI

EDU

Merge

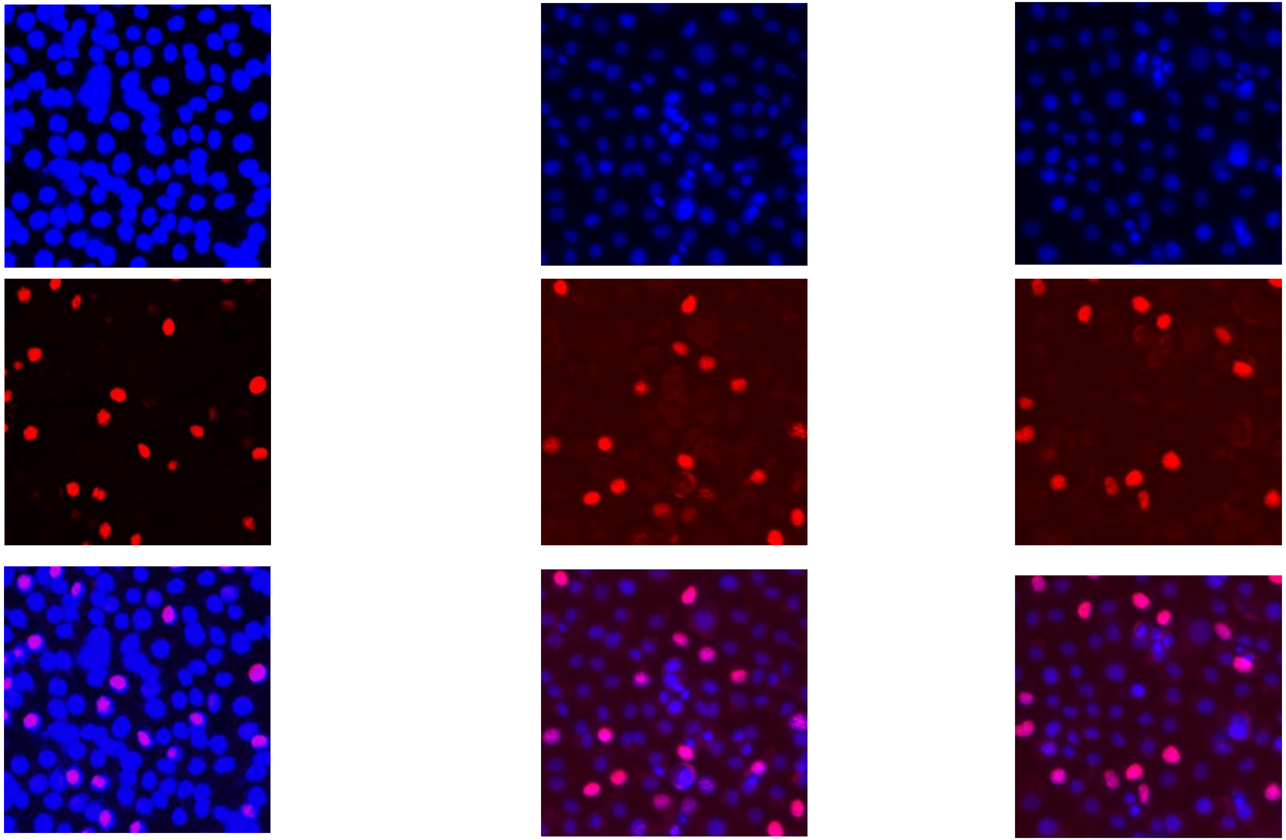

Raw data of Fig 6E

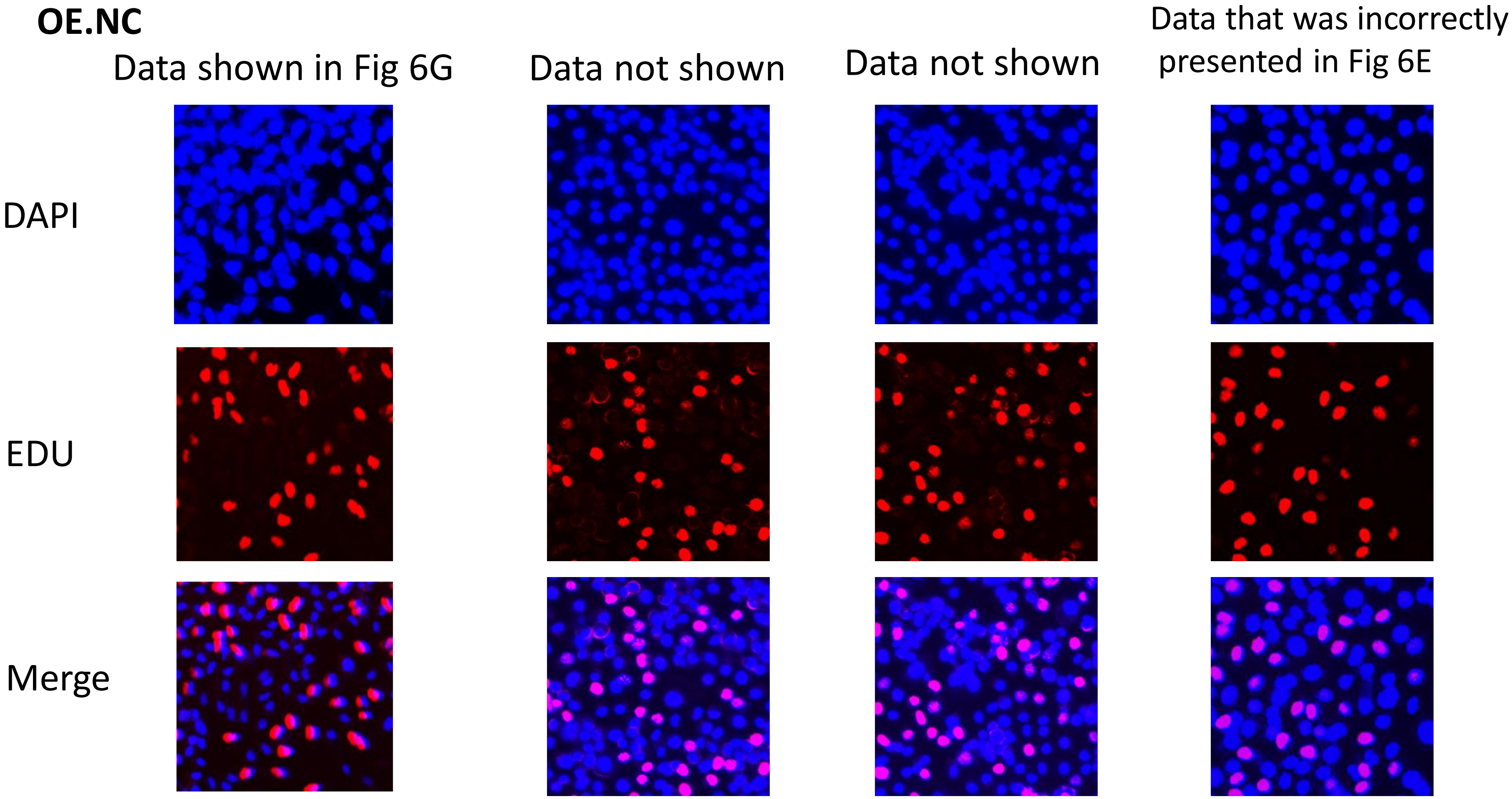

Raw data of Fig 6E

OE.HGF

Data shown in Fig 6G | Data not shown | Data not shown

DAPI

EDU

Merge

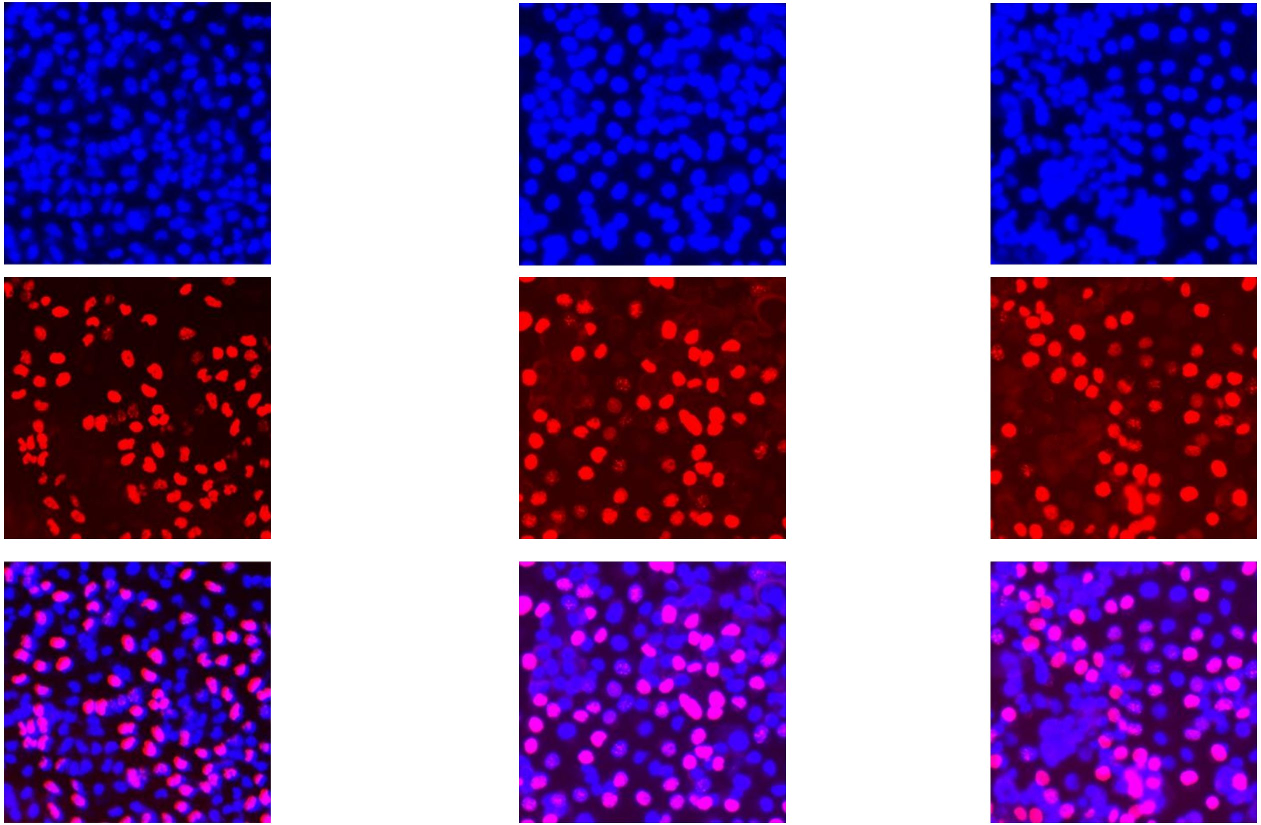

Supplement: Supplementary file 1 — Raw data [file 41467_2023_37320_MOESM1_ESM.pdf]
